# They Are What You Eat: Can Nutritional Factors during Gestation and Early Infancy Modulate the Neonatal Immune Response?

**DOI:** 10.3389/fimmu.2017.01641

**Published:** 2017-11-28

**Authors:** Sarah Prentice

**Affiliations:** ^1^Clinical Research Department, London School of Hygiene and Tropical Medicine, London, United Kingdom

**Keywords:** nutrients, immunity, ontogeny, neonatal, pregnancy, infection, supplements

## Abstract

The ontogeny of the human immune system is sensitive to nutrition even in the very early embryo, with both deficiency and excess of macro- and micronutrients being potentially detrimental. Neonates are particularly vulnerable to infectious disease due to the immaturity of the immune system and modulation of nutritional immunity may play a role in this sensitivity. This review examines whether nutrition around the time of conception, throughout pregnancy, and in early neonatal life may impact on the developing infant immune system.

## Introduction

Nearly 3 million deaths occur annually in children less than 30 days old, principally in low and middle-income countries (1). Improvements in neonatal mortality rate have proved difficult to achieve. Low-cost, easily implementable interventions are urgently needed.

Infections directly account for approximately one-third of neonatal deaths and are likely to contribute to deaths from other causes such as prematurity and in cases where babies are stillborn (1). Neonates show heightened susceptibility to infectious diseases due to a functionally immature immune system (2). Innate immune components are compromised by impaired mucosal surface integrity (3), lower levels of complement proteins (4), and reduced phagocytic capacities (5). Adaptive immune responses to pathogens are attenuated compared to adult responses, with children under 2 months old tending toward more regulatory responses with strong Th-2 and Th-17 cell polarization and weak Th-1 polarization (2, 6, 7). This is partly necessary to produce a tolerogenic environment, stopping rejection at the maternofetal interface and reducing reactions to self-antigens, and partly due to lack of primary exposure to antigens necessary to build up the adaptive immune responses. This functional immaturity of responses leaves the neonate particularly vulnerable to infectious pathogens. Decades worth of research has been directed at identifying interventions to improve neonatal immune responses to infections.

Various organs are sensitive to nutrition during embryonic and fetal development. Nutritional status can have short-term impacts on both fetal and childhood growth and development and longer term influences on adult health. Infants born following periods of nutritional deprivation, such as the Dutch Hunger Winter and identified in The Hertfordshire cohort, show increased risks of coronary heart disease, stroke, type-2 diabetes and metabolic syndrome when subsequently exposed to periods of nutrient sufficiency (8, 9). The concept that undernutrition during gestation may contribute to adult disease by having permanent effects on the structure, function and metabolism of the developing fetus, is known as the Developmental Origins of Health and Disease (DOHaD) theory. It has subsequently been shown to extend to a range of other diseases including psychiatric illnesses and cancers (10). Excess macronutrient consumption in mothers has also been associated with long-term sequelae in their offspring (11). Micronutrient deficiencies have long been known to have impacts on organogenesis, with iodine deficiency leading to congenital hypothyroidism (12) and folate deficiency increasing the risk of neural tube defects (13). Therefore, it has been hypothesized that the developing immune system is likely to be similarly sensitive to nutrition and that optimizing a mother’s nutritional state during pregnancy will have long-term benefits for the immune responses during the neonatal period and beyond.

Early human evidence that nutritional factors during gestation might specifically influence adult immune responses came from longitudinal studies carried out in The Gambia in the 1990s (14). The Gambia has a strong bimodal seasonality that has major effects on the nutritional status of the population. The dry season, running from November to June, is a time of relative nutrient security. With the previous seasons crops being harvested, macronutrients are in greater supply and manual labor levels tend to be lower. In contrast, the rainy season, running from July to October, is characterized by declining levels of food availability and higher manual labor demands as the next season’s crops are planted but the previous seasons supply is running short. This leads to deficits of both energy and micronutrient intakes that are particularly pronounced for women, who bare the brunt of much of the agricultural work (15). Analysis of demographic surveillance data available for the population from the 1940s revealed that people born during the “hungry” rainy season had a threefold higher risk of mortality from infectious diseases as adults than those born during the dry season (14). These findings were independent of subsequent nutritional status, as demonstrated by anthropometric and hematological status at 18 months of age, suggesting that the effector of these changes occurred earlier on in development. These data suggested that environmental factors, most likely nutrition, during conception, gestation and early postnatal life can have marked effects on the immune system that are stable, durable and not susceptible to modification by later improvements in nutritional status.

Nutrient intake of the mother and neonate is theoretically amenable to modification *via* supplements, which represent low-cost, easily implementable public health interventions. As such, there has been huge interest in the provision of nutritional supplements during gestation and early infancy to improve neonatal outcomes. This review summarizes the evidence regarding the impact of early life nutrition on biochemical immune markers and clinical infectious diseases outcomes in neonates.

## Potential Mechanisms for Nutritional Influences on the Developing Neonatal Immune System

Studies in older children and adults have demonstrated the important influence that different nutrients have on the immune system. These effects, and the impacts of deficiencies on susceptibility to infectious diseases, are summarized in Table 1. Although the influence of nutrients on the developing immune system *in utero* and in early neonatal life may be similar to that of older children and adults, the impact of the nutritional state of the mother on the neonatal immune system is less well described.

**Table 1:** Nutrients and their effects on immunity.

Nutrient	Effect on immunity	Effect of deficiency on clinical immune outcomes	Reference
Protein energy	*Innate*	Increased bacterial, viral, and fungal infections	(16, 17)
	Epithelial integrity		
	Complement levels		
	NK-cell activity		
	*Adaptive*		
	T-lymphocyte number and function, particularly Th1-type cytokines		
	Delayed type hypersensitivity		
	Effect on B-lymphocytes less clear		

n-3 PUFAs	Activity is largely immunosuppressant with reductions in:	Theoretical increases in inflammatory-mediated diseases and allergy. Trials suggest that supplementation reduces the risks of inflammatory-mediated diseases such as rheumatoid arthritis and improves responses to infectious disease	(18–25)
	*Innate*		
	Leukocyte chemotaxis and adhesion		
	NK-cell function		
	Innate cytokine production		
	*Adaptive*		
	T-lymphocyte signaling		

Vitamin A	*Innate*	Increased susceptibility to infections, particularly diarrhea, respiratory infections and measles. Supplementation of children from 6 months to 5 years in areas at risk of deficiency reduces all cause mortality, diarrhea incidence and mortality and measles incidence and morbidity on meta-analysis	(26–28)
	Epithelial integrity		
	Neutrophil, monocyte, macrophage, and NK-cell number and function		
	*Adaptive*		
	T-lymphocyte differentiation and migration		
	T-lymphocyte numbers, especially CD4		
	B-lymphocyte numbers		
	Antibody production and may affect the balance of production of different IgG subclasses		

B vitamins	*Vitamin B2 (riboflavin)*		(29–39)
	Phagocyte activation		
	*Vitamin B6*		
	Dendritic cell function		
	Lymphocyte maturation and growth		
	T-lymphocyte activity and delayed type hypersensitivity		
	B-lymphocyte activity and antibody production		
	*Vitamin B9 (folate)*		
	Epithelial integrity		
	NK-cell activity		
	T-lymphocyte proliferation and response to mitogenic activation		
	Cytotoxic T-lymphocyte activity		
	*Vitamin B12*		
	NK-cell activity		
	CD8+ T-cell activity		
	B-lymphocyte activity and antibody production		

Vitamin C	*Innate*	Association with increased incidence and severity of pneumonia. Supplementation in the elderly shows possible reductions in pneumonia incidence and duration	(40)
	Epithelial integrity		
	Phagocyte production		
	Antioxidative functions		
	*Adaptive*		
	T-lymphocyte maturation		
	Interferon production		

Vitamin D	*Innate*	Increased susceptibility to infections, particularly of the respiratory tract. Meta-analysis shows reduced acute respiratory tract infections when routine supplementation is given in the context of deficiency	(41–43)
	Macrophage activity (cathelecidin antimicrobial peptide expression, induction of reactive oxygen intermediaries, activation of autophagy)		
	*Adaptive*		
	T-lymphocyte number and function		
	Th1/Th2 balance skewed to Th2		
	Unclear effect on B-lymphocytes (in humans)		

Vitamin E	*Innate*	Supplementation is suggested to lead to reduced respiratory tract infections in the elderly	(37, 44, 45)
	Epithelial barrier integrity		
	NK-cell activity		
	*Adaptive*		
	T-lymphocyte proliferation and function		
	Delayed type hypersensitivity reactions		
	Vaccine-mediated antibody responses		

Zinc	*Innate*	Increased bacterial, viral and fungal infections: particularly diarrhea and pneumonia. Routine supplementation of children in at-risk areas leads to reductions in duration of diarrhea and incidence of pneumonia, in children >6 months on meta-analysis, but not in children 2–6 months old	(46–50)
	Epithelial barrier integrity		
	Proinflammatory cytokine production		
	Neutrophil oxidative burst		
	NK-cell function		
	*Adaptive*		
	T-cell maturation and proliferation		
	Th1/Th2 balance skewed to Th1		

Selenium	*Adaptive*	Increased viral virulence	(51–54)
	CD4+ T-lymphocyte proliferation and function		

Iron	*Innate*	May enhance or protect from infections with bacteria, viruses, fungi and protozoa depending on the level of iron. Although supplementation may theoretically enhance immunity to infectious diseases, untargeted supplementation may increase availability of iron for pathogen growth and virulence and increase susceptibility to, particularly, malaria and bacterial sepsis	(55, 56)
	Neutrophil, NK-cell, and macrophage activity		
	Innate cytokine production		
	*Adaptive*		
	T-lymphocyte numbers		
	No apparent effect on B-lymphocyte number and function		

Mother’s nutritional status may hypothetically affect the neonatal immune system by influencing:

- *The mother’s own immune system*: Optimizing maternal nutrition could directly enhance the neonatal immune system by increasing the quality and quantity of antibody and other immune factors available for passive transfer across the placenta and in breast milk. It could also indirectly improve neonatal immunity, by reducing the likelihood of maternal infections that may lead to preterm birth, a known cause of IgG deficiency in neonates due to reduced third-trimester antibody transfer (57). Increased maternal infections may also influence neonatal immune development *via* effects on the hypothalamic–pituitary–adrenal (HPA) axis (see below).
- *Placentation*: Maternal nutrient availability has been shown in animal and human studies to affect placentation, with affects on size, morphology, nutrient transfer receptors and vascular flow (58–63). This may theoretically affect passive transfer of antibodies and other immune factors to the fetus as well as altering the efficiency of nutrient transfer for fetal immune system development.
- *The maternal HPA axis*: The HPA axis is activated in times of low nutrient availability [particularly protein-energy malnutrition (64) and zinc deficiency (65, 66)] leading to increased circulating glucocorticoids. Increased cortisol levels can lead to both immunosuppression and altered placental function in the mother, with downstream effects for the fetus as described above, as well as directly impacting on the fetal immune system *via* actions on its own HPA axis.
- *The maternal gut microbiome*: The human intestinal tract contains more than 10^14^ bacteria and other organisms (67). These commensal microflora have evolved a complex symbiotic relationship with humans, and are increasingly recognized as essential for many aspects of human health (68). Nutrient intake influences the composition of the gut microbiota, which in turn can influence the availability of nutrients for absorption from food (69–71). The gut microbiome is crucial for the development and functioning of the mucosal immune system (72). Healthy gut flora help to promote mucosal tolerance to non-pathogenic antigens, reduce the overgrowth of pathogenic microorganisms and enhance absorption of nutrients that are potentially important for systemic immune system development (68). Dysbiosis (altered microbiome) has been associated with increased risk of immune-mediated diseases such as allergy, asthma, and inflammatory bowel diseases, as well as increased risk of infections (73). Animal models suggest that the immune development of the offspring may be influenced by the maternal microbiota in the following ways [reviewed in detail in Ref (74)]: (1) alteration of nutrient uptake having direct effects on maternal immunity and hence the availability of antibodies and immune factors for transfer to the offspring, (2) alteration of the repertoire of antibodies passively transferred to the neonate, which may alter the degree of mucosal tolerance in the neonate, and hence its own microbiome composition (75, 76), (3) bacterial metabolites derived from the microbiota may be transferred to offspring across the placenta and in breastmilk and may impact on the offspring’s developing immune system (77), and (4) organisms from the maternal microbiota can be found in placental tissue (78) and this exposure may impact directly on the developing infant immune system and indirectly by altering gestational length.

The mother’s nutritional status may also affect the neonatal immune system by directly altering the nutrients available to the developing embryo/fetus. This may theoretically have long-term effects on offspring immunity *via*:

- *Epigenetic modification*: Epigenetic modification is the process by which stable alterations to gene expression, and thus the phenotype of cells, are induced without changes to the primary DNA sequence (79, 80). These modifications may be altered in response to environmental factors, persist following cell division, and, in some cases, are heritable—providing a means by which the environment may have permanent and multigenerational impacts on phenotype (81). The three main types of epigenetic modification are (1) DNA methylation; where the degree of methylation at, primarily, CpG dinucleotide rich sites in gene-specific promoters affects the degree of expression of that gene, (2) histone modification; where the accessibility of promoter regions of genes to transcription machinery is altered by additions to protein tails, affecting the degree to which DNA transcription occurs, and (3) non-coding RNAs, where small lengths of RNA bind to target mRNA, altering its subsequent translation (81). Of these, DNA methylation has emerged as a strong candidate effector mechanism to explain the DOHaD theory as it largely occurs during embryogenesis or early postnatal life, and produces durable effects (82). Alterations in DNA methylation of key metabolic genes induced by famine exposure in early life persist for at least six decades (83, 84). Epigenetic modification could theoretically have similar long-term impacts on the expression of genes important for the immune system.
- *Organogenesis and lymphopoiesis*: The process by which organs develop during embryonic and fetal life is highly sensitive to environmental influences. It has long been known that exposure to adverse factors at critical windows of organogenesis can lead to permanent changes in organ growth and function. Development of the infant immune system is likely to be similarly susceptible to environmental influences, including nutrient levels. In older children, both the thymus and hematopoietic branches of immunity are acutely sensitive to undernutrition, with reductions in thymus size and blood cell functioning shown to occur in both acute and chronic starvation conditions (85). As both immune compartments undergo massive expansion during the gestational period, with the thymus being at its largest as a proportion of body size at birth, it is highly plausible that nutritional conditions *in utero* would impact on the neonatal immune system. Studies in animals support a link between maternal macro/micronutrient deficiency and reduced thymic size and function (86–88), which may not be fully reversible by later improvements in nutrition (89).
- *Immunoregulatory mechanisms, e.g., the neonatal HPA axis*: Maternal cortisol levels (which may be altered by nutrient availability, see above), can influence the development of the fetal HPA axis, with long-term consequences for neuroendocrine-immune interactions (90, 91). Although the developing fetus is generally protected from maternal cortisol fluctuations by the function of 11 B-hydroxysteroid dehydrogenase in the placenta, levels of this enzyme are decreased by undernutrition (92). Evidence from animal studies suggests that stimulation of the fetal HPA axis can lead to lower lymphocyte proliferation, reduced NK-cell activity, and reduced antibody responsiveness in offspring (93), as well as increasing the responsiveness of the HPA axis to stressors later in life. These effects are hypothesized to be mediated through epigenetic programming of glucocorticoid receptors (91).
- *The neonatal gut-microbiome*: The neonatal gut microbiome is strongly influenced by the maternal microbiome. Colonization of the gastrointestinal tract occurs around the time of birth (and possibly even earlier) with organisms acquired from the mother’s gastrointestinal tract, vagina, skin, and breast milk, and is influenced by delivery type, gestational age, and feeding method among other factors (94). Modification of the maternal microbiome may thus be hypothesized to influence the developing neonatal immune system both directly, by altering the neonatal microbiome composition, and indirectly, by altering the nutrient status of the mother and hence the availability of nutrients for immune system development during fetal life.

**Figure 1:**
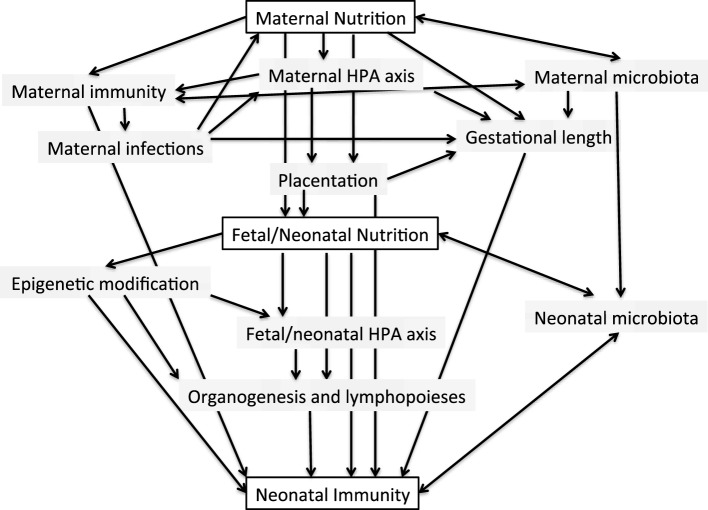
Conceptual framework for the potential interactions between maternal and early neonatal nutrition and the developing infant immune system.

A conceptual framework for the potential influences of early life nutrition on the developing infant immune system is shown in Figure 1. Evidence for such effects occurring in humans is discussed below.

## Evidence for the Influence of Pre- and Periconceptional Nutrition on the Infant Immune System

### Epigenetic Modification of the Early Embryo

Specific evidence for the impact of periconceptional nutrition on later immune functioning through epigenetic modifications has been suggested from the previously described Gambian cohort. The plasma levels of 1-carbon metabolites crucial for DNA methylation undergo seasonal variations in pregnant women. Higher levels of folate, methionine, and riboflavin, and reduced homocysteine levels occur in the nutritionally challenged rainy season (95–97). Although counterintuitive, this may be due to increased consumption of green leafy vegetables during this period, due to the need to food diversify (98). The increased level of these methyl-donor intermediaries correlates with increases in DNA methylation seen at metastable epialleles (MEs) (see Box 1) in children conceived in the rainy season (and thus born in the dry season, correlating with reduced later infectious disease mortality) (96, 99). A metastable epiallele VTRNA2-1, involved in tumor suppression and viral immunity, has been identified that is differentially methylated according to season of conception (and hence nutritional status), and is stable for at least 10 years (100). This provides the first in-human evidence that periconceptional nutrition could directly influence subsequent immune functioning. Although the clinical relevance of the variability in methylation of this ME in susceptibility to infections has yet to be proven, it provides a tantalizing suggestion that the seasonal variation in adult infectious disease mortality is mediated, at least in part, through nutritionally sensitive epigenetic modifications.

Box 1 **Metastable epialleles. A tool for investigating the influence of the periconceptional environment on offspring epigenomes.**

The inherent tissue specificity of many epigenetic changes creates challenges for the study of the influence of epigenetic modifications on adult phenotypes (99). While epidemiological association studies between gene variants and risk of disease may use easily obtainable peripheral blood draws, studies investigating epigenetic influences on disease etiology may require tissue-specific samples that are often not as accessible. Metastable epialleles (MEs) are regions of DNA where methylation is established stochastically in the early embryo and is subsequently maintained throughout all three germ-layer lineages (101). Thus, methylation of MEs occurring in the early embryonic period (pregastrulation) may be determined from peripheral blood samples.

Differential methylation of MEs in mice has been shown to have dramatic phenotypic consequences including alterations in fur color (102), tail-kinking (4, 103), and propensity to obesity (104). Methylation of murine MEs is strongly influenced by maternal nutrition and other environmental factors in the periconceptional period (105, 106). MEs in humans may have effects on adult disease and provide an easily accessible method of investigating the epigenetic pathways that may be involved in the DOHaD theory.

A number of epidemiological studies have now linked DNA methylation status at the promoter region of inflammatory mediators to nutritional status in pre- and early postnatal life (107–109), although the timing of nutritional influences causing these epigenetic modifications is difficult to prove. Methylation status of these genes has been correlated with later markers of biochemical inflammation, though effects on clinical outcomes have yet to be shown (107). Intriguingly, animal models have shown that alterations to paternal diet can alter DNA methylation in offspring, with resultant phenotypic changes increasing the risk of obesity and metabolic syndromes (110–113). The potential transgenerational influence of paternal diet on the health outcomes of offspring has also been suggested in humans from epidemiological studies carried out in Sweden. These showed a correlation between reduced food availability during the father’s, and even grandfather’s, preadolescence and increased life expectancy, with reduced risk of cardiovascular and diabetes-related mortality (114). Other studies have linked early onset of paternal obesity with increased liver enzymes and long-term changes in percentage body fat in offspring. These effects are likely to be mediated by epigenetic modification of spermatozoa, and may be sex specific (115). Thus, it may be that paternal diet is also ultimately shown to produce lasting effects on the immune system of offspring.

Although most human studies have focused on DNA methylation as a mediator of long-term effects of periconceptional environment on the health of off-spring, animal studies suggest that histone modification (116) and microRNAs (117, 118) may also play a role in the developmental origins of disease, though their importance in immune system development has yet to be investigated. Thus, it appears likely that immune system functioning is influenced by interacting and overlapping epigenetic modifications induced by nutritional status, and other environmental factors, occurring around the time of conception, during gestation and in early postnatal life.

### Placentation

Although evidence for the importance of several micronutrients including vitamin D, zinc, folate, calcium, and iron on placental growth and function exists (58, 59), studies directly investigating the effects of periconceptional maternal nutrition on placentation and subsequent fetal immunity are limited. One study that randomized non-pregnant women of child-bearing age to a multiple-micronutrient (MMN) supplementation or placebo and followed up subsequent pregnancies, showed minimal improvements in placental vascular function with MMN supplementation, but no improvements in other markers of placental function (plasminogen activation inhibitor 1 and 2 ratio) and transfer of maternal measles antibody at birth (119).

## Evidence for the Influence of Gestational Nutrition on the Infant Immune System

### Macronutrients

#### Protein Energy

The relationship between maternal nutrition and fetal growth is complex, involving maternal metabolic and endocrine, as well as placental, functioning (2, 120). However, the neonatal presentation of protein-energy malnutrition is assumed to be infants who are born small-for-gestational age (SGA). Infants born SGA or low-birth weight (LBW) have an increased risk of infectious mortality in the neonatal period and beyond (121–124). SGA/LBW infants show altered immunology, with lower complement and IgG (125), lower plasmacytoid dendritic cells, higher NK-cells and higher IgM (126), and higher inflammatory activation and T-cell turnover (127), compared to those delivered at an appropriate weight. Gambian infants born in the nutritionally deprived rainy season (a presumptive marker of reduced macronutrient supply in late gestation) show smaller neonatal thymus size (128), and have some changes to thymic function (129). These immune changes do not appear to be long lasting, however, and a seasonal effect of infectious disease incidence may contribute to these findings (130, 131). Intrauterine growth restriction has been associated with reduced vaccine responses in childhood, though inconsistently (132–135).

Given the suggested link between macronutrient deficiencies and neonatal morbidity, a number of maternal protein supplementation strategies have been evaluated (136). Balanced protein energy supplementation (containing up to 20% of energy as protein) leads to modest increases in birth weight (up to 324 g) (137), and reduces the number of SGA infants born by around a third (136). Reductions in neonatal deaths as a result of supplementation have not been clearly shown, however, with meta-analysis of the three published studies reporting neonatal mortality showing only non-significant improvements in neonatal outcomes (136, 138–140). Even if these non-significant reductions in mortality are true findings, the causal mechanisms underlying such effects are unknown, with reductions in prematurity likely to play a significant role. No clear link between maternal protein energy supplementation and improvement in neonatal immunity has been demonstrated. Maternal protein supplementation has no proven impact on later vaccine responses, mucosal immunity and delayed-type hypersensitivity reactions (130) or thymus size (141), although impacts on thymic function at the cellular level were not assessed. The lack of substantial demonstrable neonatal benefits from maternal protein energy supplementation may reflect the heterogeneous etiologies of SGA and LBW, with factors such as poor placentation and environmental toxin exposure not addressed by supplementation. It may also be due to challenges with targeting the intervention to the most at-risk subjects within populations. Subgroup analysis of supplementation studies suggest that the intervention is only beneficial when provided to malnourished individuals, and that high protein supplements may even impair fetal growth when given in the context of adequate diets (136).

#### Lipids

Maternal PUFA supplementation during gestation is associated with reductions in preterm births and small increases in birth weight (142) on systematic review. However, impacts on the immune system are less clear. Most research has been directed on the effect of fish-oil supplementation on reduction in atopy risk in offspring. Systematic reviews have suggested reductions in offspring IgE-mediated allergy and eczema following gestational/lactational n-3 PUFA supplementation, though the duration of these effects is not clear and the relative importance of the timing of supplementation during gestation or lactation is difficult to determine (143, 144). Murine studies suggest that n-3 PUFA supplementation of mothers can improve offspring responses to infections, with enhanced vaccination responses shown in mice fed high n-3 PUFA diets during gestation and lactation (145). In humans, docosahexaenoic acid (DHA) supplementation during gestation and lactation was associated with reductions in CD8+ T-cells, increases in naive CD4CD45RA+ helper cells and reductions in lymphocyte IFNγ production (146). However, this trial did not show changes to immunoglobulin levels, vaccination responses or clinical outcomes and may have been confounded by the high baseline dietary DHA levels of all participants. One trial of prenatal DHA supplementation has shown reduction in incidence and duration of cold symptoms during infancy (147). No significant evidence of reductions in neonatal outcomes such as sepsis, morbidity or mortality have been shown in systematic review of human studies, though adequately powered trials to assess these outcomes are lacking (148).

### Micronutrients

Micronutrient deficiencies are estimated to affect approximately 2 billion people worldwide. They are often particularly severe in women of reproductive age due to the high demands of pregnancy and lactation (149). Optimization of micronutrient levels in pregnant women has therefore been proposed as a strategy to enhance neonatal immunity.

#### Specific Micronutrient Supplementation during Gestation

##### Zinc

Overt zinc deficiency is now rare but moderate deficiency is common worldwide (150). Zinc supplementation of mothers leads to biochemical improvements in their zinc status and that of their offspring (151, 152). Thymus size in infants correlates with cord-blood zinc levels (153), although a recent study showed no impact of maternal zinc supplementation on infant thymic size (154). Improved hepatitis B vaccine antibody responses and delayed type hypersensitivity reactions to BCG vaccination have been shown following maternal zinc supplementation (154), but no effect on haemophilus influenza B conjugate vaccine responses has been found (155). Theses studies suggest some influence of maternal zinc supplementation on infant immune development, but the clinical impact of this is uncertain. A recent systematic review of 21 trials (>17,000 mother–infant dyads) suggests no benefit of maternal zinc supplementation for IUGR, LBW, stillbirth, and neonatal death, though small reductions in preterm birth were shown (156). No significant reduction in neonatal infective outcomes, including neonatal sepsis, umbilical infections, fever, and necrotizing enterocolitis (NEC), was seen but the number of studies reporting these outcomes was small. One study from Bangladesh showed reduced acute diarrheal and impetigo episodes in the first 6 months of life following maternal zinc supplementation, though no difference in persistent diarrhea, cough, and LRTI (157, 158). A study from Indonesia similarly reported reduced diarrheal incidence in infants <6 months old following maternal supplementation with zinc, but this was at the expense of increased episodes of cough (159). Conversely, a study in Peru did not report any benefit for diarrheal prevalence (160).

##### Vitamin D

Vitamin D deficiency is common worldwide due to lack of UV exposure in northern latitudes, darker skin pigmentation in southern latitudes, covering the skin with clothes, and vegetarian diets. There are strong correlations between maternal and umbilical cord vitamin D with deficiency or insufficiency in the mother likely to cause deficiency in offspring (161). Systematic reviews of supplementation in pregnancy suggest reduced risk of vitamin D deficiency in offspring and slight increases in birth weight (162, 163). However, no evidence for improvement in any other neonatal outcomes including neonatal mortality has been shown (162). Impacts of vitamin D deficiency on the developing immune system have been shown with reduced thymus size in offspring (164) and an association with increased CRP [although this trend is reversed with vitamin D sufficiency (>50 nmol/L) (165, 166)]. Maternal vitamin D supplementation during gestation results in increased Th1 and Th2 cytokine gene expression and reduced pattern recognition receptor expression in cord blood, following stimulation with PHA (167). Clinically, vitamin D deficiency in cord blood has been associated with increased risk of lower respiratory tract infections, wheeze, and eczema in a number of observational studies, suggesting long-term impacts on immune ontogeny, although causation is difficult to prove (168, 169). Of four studies assessing the impact of maternal vitamin D supplementation on infant risk of respiratory infections and wheeze (170–173), only one showed significant reductions in incidence of acute respiratory tract infections in offspring (170). In this study the intervention was combined with postnatal vitamin D supplements so the contribution of maternal supplementation *per se* is difficult to assess. A recent systematic review of vitamin D supplementation in pregnancy and early life did not show any reduction in the risk of persistent wheeze, eczema, or asthma, though the quality of available evidence was low (174).

##### Vitamin A

Vitamin A deficiency is associated with increased susceptibility particularly to diarrhea, respiratory infections, and measles (27). Infants born to mothers with low serum retinol had increased all-cause neonatal mortality in a study in Malawi (175). Nepali infants born to mothers with xeropthalmia (the clinical manifestation of severe vitamin A deficiency) had a 63% increased mortality within the first 6 months of life, which was reduced following maternal supplementation (176). However, large randomized controlled trials of vitamin A supplementation including more than 310,000 mother–infant pairs have failed to show benefits for perinatal and all-cause neonatal mortality on systematic review, despite reductions in maternal night-blindness and possible reductions in maternal infections (177). There is some evidence, though, that vitamin A supplementation of women may lead to long-term enhancement of natural antibody levels in offspring, perhaps acting through impacts on early lymphopoiesis (178). This suggests that long-term alterations to the neonatal immune system may occur following vitamin A supplementation, but that more sensitive outcome measures are required to identify these changes than all-cause neonatal mortality.

##### Iron

Fetal iron acquisition occurs actively across the placenta, mainly in the last trimester of pregnancy, and is highly regulated (179, 180). Direct correlations between maternal and fetal iron status are not consistently seen, as neonatal iron levels are likely to be preserved at the expense of maternal stores, but severe maternal anemia is associated with reductions in neonatal iron (181). Iron deficiency is thought to be the most prevalent micronutrient deficiency worldwide (182). It occurs particularly in low-income countries where diets tend to be low in absorbable iron and parasitic burden can be high. Systematic reviews support the use of daily or intermittent iron supplementation during pregnancy for improvement of maternal iron status and reduction in anemia (182, 183). However, no evidence for improvements in other maternal or neonatal outcomes has been found. There is a current paucity of evidence regarding specific impacts, whether beneficial or detrimental, of maternal oral iron supplementation on neonatal infection risks (184). Similarly, studies investigating a direct impact of fetal iron status on immune system ontogeny are lacking.

##### B-Vitamins, Including Folic Acid

Folate (vitamin B9) has been widely studied as a pregnancy supplement, due to its role in the reduction of neural-tube defects. A systematic review of 31 studies, mainly carried out in Europe in the 1960s and 1970s, showed a modest increase in birth weight (136 g) following maternal folate supplementation, but no reduction in preterm birth, still-birth, or neonatal death (all cause) (185). The impact of folate supplementation in pregnancy on neonatal immune parameters and infective outcomes has not been investigated. More recently, concerns have been raised that folate supplementation given beyond the first trimester, or in excessive doses during pregnancy, may be linked to an increased risk of allergy/asthma, but the evidence is largely from observational studies and is not yet conclusive (186).

Vitamin B12 deficiency is associated with an increased risk of preterm birth (187), but its supplementation in pregnancy has been little studied. One study in Bangladesh confirmed that maternal oral vitamin B12 supplementation during pregnancy and lactation led to significant increases in infant B12 levels, but this was not associated with improvements in passive transfer of influenza antibodies or levels of acute inflammation markers (188). A significant reduction in number of infants with raised CRP was shown, but the number of infants with the outcome was small and the influence of timing of supplementation during pregnancy or lactation could not be distinguished.

A systematic review of three randomized controlled trials of maternal supplementation with vitamin B6 has been shown to result in a significant reduction in mean birth weight (217 g) (189). The impact of supplementation on neonatal mortality or infections has not been studied (190).

One study of vitamin B2 supplementation during pregnancy and lactation exists, which showed modest increases in infant riboflavin levels, but did not report neonatal outcomes (191). Sole supplementation with other B-vitamins has not been studied in the context of pregnancy and their impacts on the developing neonatal immune system are unknown.

##### Other Vitamins and Trace Elements

A number of other micronutrients with known immunomodulatory effects in adults have been little studied in neonates. Longitudinal studies of the influence of maternal diet on infant respiratory outcomes have suggested inverse associations between maternal vitamin E intake and infant asthma/wheeze (192–194), however, this has not been borne out in randomized controlled trials of maternal supplementation (195). Maternal selenium deficiency leads to low selenium status of neonates and is associated with reduced circulating adaptive immune cells and *in vitro* thymocyte activation (196). Observational studies have associated maternal selenium deficiency with enhanced risk of infant infections in the first 6 weeks of life, but these studies are at high risk of confounding (197). One supplementation study of selenium in HIV positive mothers showed a possible reduced risk of all-cause child mortality after 6 weeks of life, but a non-significant increase in fetal deaths (198). No studies have yet investigated maternal vitamin C, vitamin E, or selenium supplementation for neonatal immune outcomes specifically. There is also no current evidence for reductions in the more gross markers that may be associated with neonatal immune function (IUGR, LBW, preterm birth, perinatal, or neonatal death) from supplementation in pregnancy of vitamin C (199), vitamin E (200), copper (201), or selenium (198).

#### Multiple Micronutrient Supplementation during Gestation

When micronutrient deficiencies exist they are often multiple, due to poor quantity and diversity of available foodstuffs (149). Identification and targeted treatment of specific deficiencies in pregnant women is expensive and programmatically challenging. Therefore many studies aiming to enhance micronutrient levels in pregnancy use multiple micronutrient (MMN) supplements that provide the recommended daily allowance of all vitamins and minerals in one tablet (202). However, the evidence supporting the use of MMNs for neonatal outcomes in general, and neonatal immunity specifically is not clear. Meta-analysis of studies involving more than 135,000 women showed modest increase in birth weight (22–54 g), with corresponding reduction in babies born SGA or LBW, following MMN supplementation compared to standard iron and folic acid supplementation (203). These improved birth outcomes did not translate into improvements in neonatal and infant morbidity/mortality including from infectious disease (204). No MMN supplementation studies to date have investigated neonatal immune parameters specifically, although one randomized controlled trial from The Gambia is due to report shortly (205).

### Probiotics, Prebiotics, and Synbiotics

Studies of maternal supplementation with probiotics (live microorganisms that contribute to a “healthy” gut microbiota), prebiotics [nutrients that promote growth of healthy bacteria, such as non-digestible oligosaccharides (206)], and synbiotics (a combination or pro- and prebiotics), for modulation of the neonatal immune system have been conducted in humans, but are relatively limited. A number of randomized controlled trials have shown that maternal consumption of probiotics or synbiotics can lead to measurable changes in the composition of their offspring’s microbiome (207–210) and to changes in immune markers in the mother (211). However, alterations in infant immune markers following maternal supplementation, such as vaccine responses and cytokine levels, have been harder to show (212). Reduced incidence of eczema, though not asthma and wheeze, in infants has been suggested from systematic reviews of trials of prenatal supplementation but the effects may not be durable (72, 213–216). One small trial has shown reduced gastrointestinal infections in infants born to mothers supplemented with probiotics (211), and another a reduction in respiratory infections (217), but these findings need to be confirmed in larger studies.

## Evidence for the Influence of Early Postnatal Nutrition on the Infant Immune System

The major nutritional influence on neonatal immunity is breast milk, which contains immunological components such as antibodies, anti-inflammatory cytokines and other antimicrobial factors, as well as the macro and micronutrients to support neonatal immune system development (218). Its benefits over formula milk for protection against various infections, atopy, and allergy are well reviewed elsewhere (219, 220). Here, we focus on the potential impact of supplementary nutritional interventions for the breastfeeding mother and neonate on the developing neonatal immune system.

### Lactational Supplementation

The composition of breast milk is highly regulated according to the neonate’s needs with the concentrations of many components maintained independently of maternal nutritional status and diet (221). Some immunomodulatory micronutrients, such as iron, folate and zinc (222, 223) and macronutrients such as arachadonic acid (224, 225) are not altered in the breast milk according to maternal diet. Therefore, maternal supplementation of these nutrients would likely have little or no impact on neonatal immune outcomes and they are not discussed further in this section. However, some immunoactive nutrients in breast milk are impacted by diet and their concentrations in milk vary worldwide. These include vitamin A, vitamin D, B vitamins, selenium, and PUFAs, particularly DHA (34, 221).

#### Micronutrient Supplementation of Lactating Mothers

##### Vitamin A

Vitamin A is not only necessary for the developing neonatal immune system, its presence in breast milk is also important for the regulation of a number of breast milk proteins important for host defense (226). Infants are born with low vitamin A stores in the liver, and breast milk is the main source of vitamin A for infants during the first 6 months of life (227). Numerous reports have shown decreased breast milk vitamin A concentration with maternal deficiency, and increased concentrations with high exogenous vitamin A levels (228, 229). However, the results of postnatal maternal vitamin A supplementation studies for neonatal outcomes have been inconclusive. Systematic reviews of both lower dose (200,000 IU) and higher dose (400,000 IU) postpartum maternal vitamin A supplementation have shown only small increases in breast milk retinol concentrations (230) and a lack of supporting evidence for reduced infant morbidity (including from infections) to 6 months of age (230, 231). As a result, WHO no longer recommends routine postpartum vitamin A supplementation for women in low- and middle-income countries (WHO 2017). Studies on the effects of postpartum vitamin A supplementation on immunological outcomes specifically are limited and inconclusive. Studies variously report increases and no change to sIgA following postpartum vitamin A supplementation (226, 232). Further studies looking at a wider array of immunological parameters, and altering the timing of vitamin A supplementation are ongoing (226).

##### Vitamin D

Vitamin D deficiency is relatively common in breastfed infants, with low concentrations in milk even from vitamin D sufficient mothers (233). Studies investigating maternal postpartum supplementation have shown variable results, though on balance suggest supplementation may enhance infant vitamin D status (234–238). At present, however, direct neonatal supplementation of with vitamin D is the preferred method of enhancing neonatal vitamin D status (see below). Studies investigating the impact of vitamin D supplementation in breast-feeding women for neonatal immunological outcomes are lacking.

##### B-Vitamins

B-vitamins levels in the breast milk are largely amenable to improvements with supplementation of the mother (with the exception of folate) (34, 239), but there are no studies looking at the impact of lactational B-vitamin supplementation on neonatal immune outcomes.

##### Selenium

Selenium levels in breast milk are sensitive to dietary intake (240) and can be increased by supplementation (240, 241) [although these effects have not been consistently shown (197, 242)] and alter infant selenium status (243). Although selenium deficiency in infants has been associated with increased risk of respiratory infections in the first 6 weeks of life (197), large studies investigating maternal postpartum selenium supplementation for infant infectious morbidity have not been conducted.

##### Multiple Micronutrients

Given the high prevalence of coexisting micronutrient deficiencies world-wide, there is a surprising lack of studies investigating the impact of multiple micronutrient supplements in breastfeeding mothers for infant outcomes (34). Only two small trials (52 women total) have compared MMN supplementation with nothing/placebo in breast feeding mothers, and reported on neither infant morbidity nor immunological outcomes (34, 232).

#### Lipid Supplementation of Lactating Mothers

The concentration of PUFAs, particularly DHA, in breast milk is highly affected by maternal diet (244), and PUFA supplementation increases levels in breast milk (245). Breast milk n3:n6 ratios have been associated with risk of allergy and atopy in infants in observational studies (246–248) although not consistently (249). Fish oil supplements provided during lactation alter cytokine production in the infant for at least 2.5 years, favoring faster immune maturation and Th1 polarization (250). Given the increasing existence of imbalanced n3:n6 ratios in westernized diets, there has been interest in providing PUFA supplements to lactating women for allergy prevention in infants, although concerns exist about potential negative impacts on infectious disease susceptibility (251, 252). However, at present only two studies (667 participants) have investigated postnatal maternal PUFA supplementation specifically, and although persisting alterations in cytokines have been shown, the studies were underpowered to detect differences in infant atopic disease or infectious morbidity (143, 250).

#### Probiotic, Prebiotic, and Synbiotic Supplementation of Lactating Mothers

Supplementation of lactating mothers with probiotics has been associated with alterations to breast milk cytokines and infant fecal IgA (253), and changes to the breast milk and infant microbiomes (254). Studies supplementing mothers with probiotics during lactation suggest a reduced risk of dermatitis, but interventions tended to combine pre- and postnatal supplementation, so the specific impact of lactational supplementation is difficult to determine (255). As with prenatal maternal supplementation, effects on infant immune outcomes following lactational supplementation require further evaluation (72, 256).

### Neonatal Supplementation

Direct supplementation with crucial nutrients in the neonatal period has also been assessed as a strategy to protect infants from deficiency. However, in the majority of cases, despite improvements in the nutrient status of infants, no clear evidence for improvements in clinical or biochemical immune outcomes has been shown.

#### Micronutrient Supplementation of the Neonate

##### Zinc

Zinc use in older infants has been associated with reductions in diarrhea duration (48) and lower respiratory tract infections incidence (47), but results following supplementation in the neonatal period have been more equivocal (257–261). One small study of zinc supplementation as an adjunct to antibiotics in neonates with sepsis showed a reduction in treatment failures and a non-significant 43% reduction in mortality (262). A larger study to investigate this is currently ongoing (263). Studies directly investigating the impact of neonatal zinc supplementation on immunological markers are limited. Routine zinc supplementation has not been associated with improvements in OPV seroconversion rates (264), although its use as an adjunct to antibiotics in neonatal sepsis has been associated with significantly reduced serum calprotectin, IL-6, and TNFα and a non-significant reduction in mortality (265).

##### Vitamin D

Vitamin D supplementation is recommended routinely in many countries for its impact on calcium and bone metabolism, but large-scale evidence for postnatal supplementation on any immunological disease outcomes (infection or allergy) is lacking (266). A recent systematic review of supplementation in children below 5 years of age did not show reductions in diarrhea and pneumonia incidence despite raised vitamin D levels in supplemented children, though supplementation in the neonatal period was not looked at specifically (42). One trial of maternal and infant vitamin D supplementation has suggested lower numbers of respiratory infection primary care visits following high dose maternal and infant supplementation, compared to low dose (170). A large trial to investigate immunological outcomes following neonatal vitamin D supplementation in breastfed infants is currently underway (266).

##### Vitamin A

Vitamin A supplementation in children from low- and middle-income countries aged 6 months to 5 years is associated with reductions in all-cause mortality of around one-third on systematic review (28). In contrast, a large systematic review of trials including more than 168,000 infants from low- and middle-income countries did not show any benefit of vitamin A supplementation when given in the neonatal period (267). Effects of supplementation may differ by underlying vitamin A status of the population, as reductions in all-cause mortality were suggested in the South Asian studies but not in the African studies. The African studies also showed concerning side-effects with increased transient bulging of the fontanelle and interactions of vitamin A with routine immunizations, particularly in female infants (268, 269). Studies investigating the effects of neonatal vitamin A on immunological parameters are limited. One study conducted in Guinea Bissau showed no effect of neonatal vitamin A supplementation on BCG vaccination responses at 6 months of age (270), although some evidence of reduced TNFα and IL-10 production in girls who have not received DTP vaccination (271). Two RCTs are currently ongoing to specifically investigate the effects of neonatal vitamin A supplementation on the immune system, but these have yet to report (226, 272). Routine vitamin A supplementation in children below 6 months of age is not currently recommended.

##### Iron

The provision of iron supplements to neonates deserves special mention due to its potential for increasing susceptibility to infections by enhancing iron availability for pathogens (55). Studies conducted in the 1970s showed that injecting neonates with iron dextran at birth significantly increased the risk of *Escherichia coli* meningitis and sepsis (273) and enhanced *in vitro* bacterial growth (274, 275). This may have been partly due to the mode of delivery, as parenteral iron administration is not subject to regulated uptake in the gut and therefore may overwhelm iron homeostatic mechanisms in iron replete children, but similar concerns exist with the untargeted provision of oral iron supplements. Older children given iron supplements from 4 months of age have increased risk of gastrointestinal infections (276), adult studies show increased *in vitro* bacterial growth in serum after oral iron supplementation (277) and there are suggestions that malaria risk is increased when oral iron is provided to iron replete children in endemic countries (55, 278). Human breast milk contains low levels of iron and has specific iron chelating agents such as lactoferrin. Our group and others have also shown that serum iron drops rapidly and profoundly in the first 12 h of life that and persists at low levels for at least 4 days. This low serum iron is associated with reduced *ex vivo* bacterial growth (279, 280). Taken together, this evidence suggests that humans may have evolved to mitigate against the enhanced pathogen susceptibility and oxidative stress that results from high iron loads. Therefore provision of exogenous iron to the neonate, except in specific situations where severe iron deficiency anemia has been diagnosed, may do more harm than good. In fact, there is increasing interest in novel therapeutics, such as lactoferrin and hepcidin agonists, that reduce serum iron in the context of neonatal infections (281–283). However, as preterm and growth-restricted infants have lower iron stores from birth, routine iron supplementation is often given, starting from 4 weeks of age, in high-income countries (284). In these settings, where infectious disease burden is low, no adverse infective outcomes have been shown on systematic review (285).

##### Other Vitamins and Trace Elements

Parenteral selenium supplementation of very LBW infants in NICU has been shown to increase selenium levels and reduce the incidence of neonatal sepsis, but systematic review of available evidence does not show improvements in survival (286, 287). No similar studies of oral supplementation in normal weight, term, breastfed infants in areas of selenium deficiency have been conducted. Studies looking at the effects of neonatal selenium, B-complex vitamins, vitamins C and E, or combined micronutrient supplements on immunological parameters specifically are lacking.

#### Probiotic, Prebiotic, and Synbiotic Supplementation in the Neonate

Interest in the provision of probiotics, prebiotics, or synbiotics directly to neonates that are at risk of dysbiosis of the gut microbiome has exploded in recent years (255). Preterm infants are at particular risk of dysbiosis, not only due to gut immaturity, but because they often have reduced or delayed enteral feeds and increased exposure to antibiotics. Failure to establish normal gut flora is linked to higher risk of NEC and nosocomial sepsis (288). Systematic review of studies providing probiotics to low-birth weight infants in neonatal units, suggest a reduction in grade II or III NEC and all-cause mortality, though no significant reductions in sepsis (289, 290). Not all studies have shown clear benefits for NEC, however, and multistrain probiotics appear more beneficial than single strain organisms (291). Prebiotic supplements have not been shown to result in significant reduction in NEC, all-cause mortality or sepsis when given to preterm infants (292). The long-term health implications of use of pre- and probiotic supplements in preterm infants are not currently known. Provision of probiotics and prebiotics to formula fed infants, in attempts to produce a gut microbiome profile similar to breastfed infants, has also been extensively studied. Although beyond the scope of this review, these studies suggest reductions in atopic disease (though few studies have follow-up of sufficient duration to assess long-term effects) (293) and some limited evidence on systematic review for reductions in gastrointestinal and respiratory infections (294, 295). More excitingly, a recent randomized controlled trial in breastfed infants in rural India showed that synbiotic administration during the first 7 days of life led to a 40% reduction in sepsis and all-cause mortality in the first 60 days of life (296). This suggests that in certain situations even the breastfed microbiome may be altered for immunological benefits in the early neonatal period. However, further studies to examine the effect of different strains, dosages and durations, as well as the long-term consequences of synbiotic administration, will be needed before synbiotics could be considered as a public health intervention for neonatal sepsis.

## Summary

Despite multiple animal and human studies associating nutrient deficiencies with adverse immunological outcomes, there is strikingly little evidence to suggest nutritional supplementation during gestation and early infancy has benefits for neonatal responses to infection or allergic disease prevention.

There are a number of plausible explanations for the lack of significant and consistent impacts of individual or combined nutrient supplements on neonatal outcomes. First, it may reflect the heterogeneity of the studied populations in-terms of their underlying nutritional status. Improvements in clinical outcomes are likely to be most where deficiencies are highest. The transfer of many nutrients across the placenta, such as vitamin A (177) and iron (179), occurs actively and is regulated by the fetus, meaning that even in the context of maternal insufficiency the fetus remains relatively protected. As a result, maternal supplementation might only benefit infants born to mothers with critical deficiencies. Large population studies including non-deficient participants will have reduced power to detect clinical benefit. Maternal vitamin A supplementation, for instance, had larger effects on maternal and neonatal outcomes in Nepal (297), where severe deficiency is common, compared to Ghana (298) and Bangladesh (299) where levels of deficiency are more moderate (177). Second, in many studies iron and folate were provided to mothers in the non-intervention arm. As these can also impact on neonatal infective outcomes, this may have confounded the results (156). Third, the optimal level of supplementation of micro- and macronutrients for neonatal outcomes is not known and dosages often differ between studies (300). Micronutrients have nutrient–nutrient interactions that may alter the availability of other immunity modulating nutrients and have a rate-limiting effect on immune development (301). High levels of iron, zinc, and protein, for instance, can have counterintuitively negative effects on the immune system, and may have detrimental outcomes when given to sufficient women (302). If this is the case, then population-based treatment as a public health intervention becomes challenging and less measurably effective. Fourth, it may be that the onset of maternal supplementation in the studies was too late in gestation to have lasting effects on immune system development. Supplementation was commenced after 12 weeks of age in many studies, which would miss an early programming effect of nutrients if one exists. As a number of supplementation studies reported improvements in mothers nutrient status following supplementation, but no improvements in clinical outcome for the offspring, it would be interesting to know whether this enhanced nutritional status had positive impacts on future pregnancies, by improving nutrient status during the periconceptional period. Lastly, despite the large number of studies investigating maternal nutrient supplementation, those designed specifically to look at the effects on neonatal immune development and infectious/allergic disease outcomes are limited and further research with more sensitive outcome markers is warranted.

Although the evidence for the benefits of nutritional supplements in pregnancy and early infancy has so far been disappointing, some exciting possibilities remain. The persisting epigenetic changes induced by nutritional factors around the time of conception, which may impact on immune functioning in later life, warrants further study to assess their impact on neonatal infections, allergy and the amenability to supplementation. The potential benefit of probiotics and synbiotics for infectious disease and allergic outcomes in infancy is also extremely exciting. The World Allergy Organisation has recently recommended probiotic use during gestation, lactation and early life for infants at high risk of atopic disease (303), but further work to determine the most effective strains, dosage and duration, and whether these vary by geographical region, will be needed before their widespread use as a public health intervention against neonatal infections can be recommended.

## Author Contributions

SP was responsible for all parts of this article.

## Conflict of Interest Statement

The author declares that the research was conducted in the absence of any commercial or financial relationships that could be construed as a potential conflict of interest. The reviewer DM and handling editor declared their shared affiliation.
